# Effective diagnosis by real-time PCR of herpes simplex diffuse endotheliitis that is similar in appearance to fungal keratitis: case series

**DOI:** 10.1186/s12348-021-00250-6

**Published:** 2021-07-12

**Authors:** Daisuke Todokoro, Mayumi Hosogai, Satoko Nakano, Hideo Akiyama

**Affiliations:** 1 Department of Ophthalmology, Gunma University Graduate School of Medicine, 3-39-15 Showa-machi, Maebashi, Japan; 2 Department of Ophthalmology, Oita University, Yufu, Japan

**Keywords:** Herpes simplex keratitis, Diffuse endotheliitis, PCR, HSV-1, Fungal keratitis

## Abstract

**Purpose:**

Herpes simplex diffuse endotheliitis with accompanying feathery infiltration is difficult to diagnose due to corneal findings that are similar to fungal keratitis. This case series reports on the effectiveness of using real-time polymerase chain reaction (PCR) to diagnose herpes simplex diffuse endotheliitis that is similar in appearance to fungal keratitis.

**Methods:**

After extracting corneal smear sample DNA, samples were then applied to two independent PCR assays, a qualitative multiplex 24-pathogen strip PCR assay, and a quantitative real-time PCR assay of herpes simplex virus type 1 (HSV-1).

**Results:**

All 3 cases showed ciliary injection, feathery infiltration in the corneal stroma and hypopyon, which are corneal findings similar to that observed for fungal keratitis. Retrocorneal plaques, which showed clear boundaries between the corneal endothelial surfaces and retrocorneal plaques in anterior segment optical coherence tomography, were observed in 2 out of 3 cases. Corneal scraping was performed in all cases, followed by initiation of antifungal treatment. However, real-time PCR of the corneal scraping detected 6.0 × 10^6^, 1.0 × 10^5^ and 5.0 × 10^5^ copies/μg glyceraldehyde 3-phosphate dehydrogenase (GAPDH) of HSV-1 DNA per each microgram of the samples. Fungi were not cultured in any of the cases. After switching the medication from antifungal to antiviral, the feathery corneal infiltration was cured with only mild scarring.

**Conclusions:**

Real-time PCR was an effective tool in diagnosing HSV diffuse endotheliitis with feathery infiltration. Topical corticosteroids in conjunction with oral and topical antivirals were an effective treatment.

## Introduction

Herpes simplex keratitis (HSK) presents various clinical manifestations including, infectious epithelial keratitis, neurotrophic keratopathy, necrotizing stromal keratitis, immune stromal keratitis and endotheliitis [1]. Although the diagnosis of typical cases can easily be made based only on clinical findings, the diagnosis of atypical or complicated cases is often challenging, even for experienced ophthalmologists. Among the various HSK clinical manifestations, the diagnosis of diffuse endotheliitis with accompanying feathery infiltration is challenging, as it is similar in appearance to fungal keratitis. However, the treatments for HSK and fungal keratitis are completely different, as corticosteroids are necessary for treating stromal or endothelial immune reactions associated with HSK.

Recently, real-time polymerase chain reaction (PCR) has been utilized to diagnose various atypical cases of HSK [2–4]. However, details on the use of real-time PCR as a diagnostic tool for diffuse endotheliitis have yet to be reported, as it is a rare form of endotheliitis. In the current report, we present 3 cases of herpes simplex diffuse endotheliitis that were found to be very similar to fungal keratitis and which were successfully diagnosed through the use of real-time PCR.

## Case description

### Case 1

**Fig. 1:**
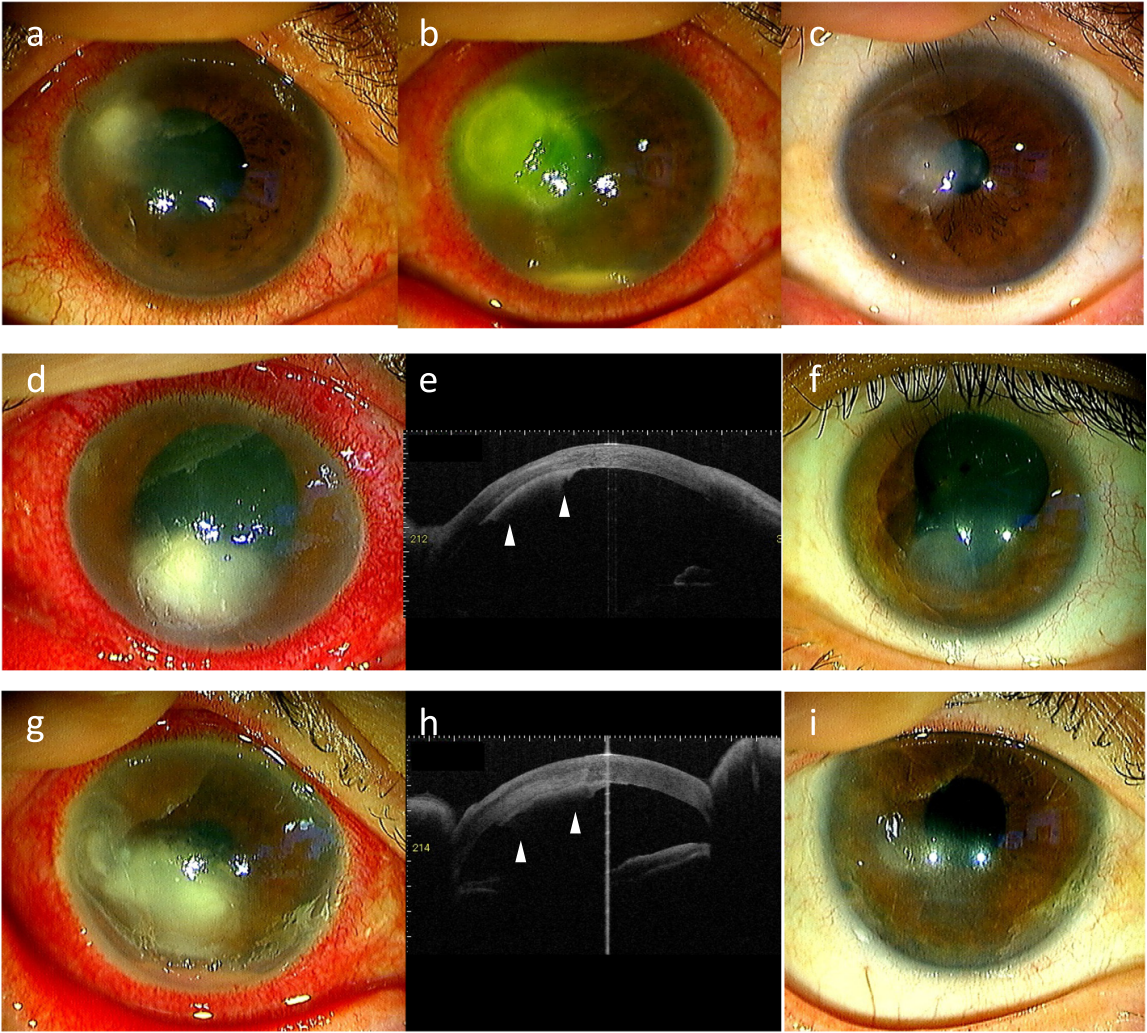
Slit-lamp and anterior segment optical coherence tomography (ASOCT) photographs obtained in each of the cases. Case #1: The feathery infiltration observed at the first visit (**a**) expanded to the central cornea and showed a ring-shaped lesion and hypopyon (**b**). At 2 months, there was keratitis scarring observed (**c**). Case #2: Full-thickness feathery infiltration (**d**) and ASOCT scanning of the retrocorneal plaque (**e**, arrowheads) performed at the first visit. At 2 months, keratitis scarring was observed (**f**). Case #3: Deep stromal feathery infiltration (**g**) and ASOCT scanning of the retrocorneal plaque (**h**, arrowheads) performed at the first visit. At 2 months, keratitis scarring was observed (**i**)

One month after a healthy 60-year-old male carpenter had an accident in which a foreign body entered his left eye while using a grinder, the patient noticed blurred vision of his left eye and visited a nearby hospital. The initial doctor who examined the patient suspected bacterial keratitis and prescribed levofloxacin and cefmenoxime eye drops. Due to the lack of improvement after 1 week in conjunction with negative bacterial culture results, he was referred to our department. Best corrected visual acuity (BCVA) at his first visit was 1.2 (right) and 0.04 (left). Intraocular pressures (IOPs) were within normal limits in both eyes. Slit-lamp examination of the left eye showed ciliary injection, cells in the anterior chamber and feathery infiltration in the upper nasal peripheral cornea with fibrin formation on the corneal endothelium (Fig. 1a). Small keratic precipitates (KPs) were seen in the lower cornea. The findings for the right eye and the fundus were normal. As we suspected fungal or bacterial keratitis, we performed corneal scraping. However, direct microscopy did not find any pathogens, including bacteria or fungi. Although we continued topical antibiotics, the feathery lesion expanded to the central cornea and exhibited a ring-shaped lesion with slight hypopyon after 2 weeks (Fig. 1b). Results for the Cochet-Bonnet esthesiometer were 60 and 50 mm in the right and left cornea, respectively. Even though no growth was observed in a bacterial and fungal culture, we clinically suspected a fungal infection and thus, we added hourly topical voriconazole 1% and miconazole 0.1%. Subsequently, we then performed corneal scraping again for multiplex real-time PCR [5]. At 3 weeks after his first visit, PCR detected 6.0 × 10^6^ copies/μg glyceraldehyde 3-phosphate dehydrogenase (GAPDH) of the herpes simplex virus type 1 (HSV-1) DNA per microgram of sample. As a result, we switched the patient from antifungals to topical corticosteroids (fluorometholone 0.1%, twice a day) and oral valacyclovir 500 mg twice daily for 5 days (1000 mg total daily dose). Although there was gradual scarring due to the keratitis, the left BCVA improved to 1.2 at 2 months after his first visit (Fig. 1c).

### Case 2

After a 52-year-old healthy man with herpes keratitis was treated with topical steroid at a nearby eye clinic, he was referred to our department due to the development of feathery infiltration in his left cornea. BCVA at his first visit was 1.2 (right) and hand motion (left), respectively. IOP of the right eye was 20 mmHg, while the left eye was elevated to 45 mmHg. Slit-lamp examination of the left eye showed ciliary injection, and full-thickness feathery corneal infiltration with round epithelial defect (Fig. 1d). In addition, anterior segment optical coherence tomography (ASOCT) demonstrated the presence of a retrocorneal plaque (Fig. 1e). After performing corneal scraping, direct microscopy did not find the presence of any pathogen. The residual sample was then evaluated by culture and multiplex real-time PCR [5]. Since we clinically suspected fungal keratitis, the patient was started on topical voriconazole 1% hourly, levofloxacin 1.5% 6 times per day, atropine 1% once per day and oral acetazolamide. After 1 week, there was no improvement in the corneal findings, and a hypopyon developed. As PCR detected 1.0 × 10^5^ copies/μg GAPDH of HSV-1 DNA, we added topical acyclovir 3% ointment, 5 times per day and oral valacyclovir 500 mg twice daily for 5 days. At 2 weeks after his first visit, there was no growth observed in his fungal culture. Based on these results, his antifungal was stopped, and topical betamethasone 0.1% twice a day was added. Small KPs were observed around the lesion during the resolution of the corneal edema. After 2 months, there was scarring of the lesion, with the BCVA improving to 0.2 (Fig. 1f). There has been no recurrence observed for 1 year.

### Case 3

A 76-year-old healthy woman was being treated with eye drops that included topical fluorometholone 0.1% for dry eye syndrome. After developing pain in her right eye, she visited a hospital. The examining doctor treated her for 2 weeks with topical antibiotics and natamycin 1% ointment and oral itraconazole. However, as there was no improvement in her right eye, she was referred to our department. At her initial visit, BCVAs were hand motion (right) and 1.2 (left), respectively. IOPs were within normal range in both eyes. Slit-lamp examination showed deep stromal feathery infiltration in the right cornea with retrocorneal plaque, hypopyon and iris rubeosis (Fig. 1g, h). Corneal scraping and a subsequent direct microscopy evaluation did not show either bacteria or fungi. The residual samples were then evaluated by culture and multiplex PCR [5]. Even though we did not detect fungi, we clinically suspected fungal keratitis and therefore started treatment with topical voriconazole 1% hourly, levofloxacin and cefmenoxime 6 times per day. However, while real-time PCR detected 5.0 × 10^5^ copies/μg GAPDH of HSV-1 DNA on the fifth day after his first visit, there was no pathogen observed in the culture. As a result, we then tapered the antifungals and added topical acyclovir 3% ointment 5 times a day along with fluorometholone 0.1% twice a day and oral valacyclovir 500 mg twice daily for 5 days. After the treatment, KPs were seen around the lesion and there was corneal scarring observed. At 2 months after the treatment, the BCVA of the right eye had improved to 1.0 (Fig. 1i). There has been no recurrence observed for 6 months.

### Real-time PCR of HSV-1

DNA of the corneal smear samples was extracted using a QIAamp DNA Mini Kit (Qiagen, Hilden, Germany) in accordance with the manufacturer’s protocol. The DNA was eluted with 40 to 100 μL elution buffer and quantified with NanoDrop 1000 (Thermo Fisher Scientific, Inc., Waltham, MA, USA).

The genomic DNA of HSV-1 was measured using two independent PCR assays that included a combination of a qualitative multiplex PCR using the 24-pathogen strip PCR assay [5], and a quantitative real-time PCR. The 24-pathogen strip PCR, which comprehensively detects 24 ocular infectious pathogens including HSV-1, HSV-2, varicella-zoster virus, adenovirus, *Mycobacterium tuberculosis*, bacterial 16S rRNA, *Candida* species, *Aspergillus* species, *Fusarium* species, fungal 18S rRNA, *Acanthamoeba*, and other pathogens was performed as previously described [5]. PCR reaction conditions were 95 °C for 10 s, followed by 45 cycles at 95 °C for 5 s, and at 60 °C for 60 s. GAPDH was also used as a PCR monitoring control. Semiquantitative measurement with quantification cycle values after PCR provided an indication of the approximate amount of pathogen.

The real-time PCR assay was performed to quantitatively measure the genomic DNA of HSV-1. This experiment used GAPDH as an internal positive extraction and amplification control. Each PCR reaction (20 μL total volume) contained 10 μL buffer, 7.2 μL PCR-grade water, 2 μL DNA or DW (negative control), 0.6 μL primer-probe mix, and 0.2 μL enzyme. The calibration curves were generated using positive control DNA dilutions (10^6^, 10^4^ and 10^2^ copies/μL). Primers and probes for HSV-1 have been previously described [6]. The products were subjected to 45 cycles of PCR amplification, with cycling conditions set at 95 °C for 10 s, followed by 45 cycles at 95 °C for 5 s, and at 60 °C for 30 s. Mx3000P qPCR system (Agilent Technologies, Santa Clara, CA, USA) was used for both of the PCR assays. A copy number of greater than 50 copies/mL was used to define the clinical positivity as per a previous report [7].

## Conclusions

Holland et al. previously classified HSV endotheliitis into three forms: disciform, diffuse or linear endotheliitis [1, 8]. HSV diffuse endotheliitis has been described as typically having scattered KPs over the entire cornea with overlying diffuse stromal edema that may be accompanied by dense retrocorneal plaques of inflammatory cells with hypopyon in severe cases [1]. In our current study, the clinical courses in all cases showed hypopyon, with 2 cases showing retrocorneal plaques. However, KPs at the first examination were only seen in Case #1 (Table 1). There was no neovascularization into the corneal stroma observed in any of the cases. In addition, all of the patients were immunocompetent, with only Case #2 having a history of HSK. All of the patients responded well to topical steroids, with the feathery corneal infiltrations successfully cured with only mild scarring. After addition of topical steroids, Cases #2 and #3 developed small KPs during resolution of the corneal edema. Overall, we believe that these 3 cases all belong to the clinical entity of HSV diffuse endotheliitis.

**Table 1:** Summarized results of ophthalmologic examinations

	Case #1	Case #2	Case #3
Increased IOP	–	+	–
Decreased corneal sensation	–	NT	NT
Feathery infiltration	+	+	+
Retrocorneal plaque	−*	+	+
Hypopyon	+	+	+
Keratic precipitates	+	–	–
Iris rubeosis	–	–	+
Copy number of HSV-1 DNA	6.0 × 10^6^	1.0 × 10^5^	5.0 × 10^5^

*Only fibrin formation on the corneal endothelium

The diagnosis of HSV endotheliitis with accompanying feathery infiltration is difficult, as the clinical findings for this are highly similar to that observed for fungal keratitis. Based on our current findings, we propose that real-time PCR of corneal scraping samples are an essential diagnostic tool in the elucidation of this disease. Indeed, HSV-1 DNA of more than 1.0 × 10^5^ copies/μg GAPDH was detected in all of the cases in this study, which was much higher than the copy numbers that are obtained in normal individuals [9]. In addition, direct microscopy and ASOCT are also useful as an auxiliary diagnosis. Moreover, direct microscopy of corneal scraping should also be done in order to demonstrate the absence of fungal elements and exclude fungal keratitis. ASOCT has been shown to be effective for evaluating the tomographic features of retrocorneal plaques. Takezawa et al. reported that ASOCT evaluation of endothelial plaques of bacterial or herpetic keratitis demonstrated there were clear boundaries between the corneal endothelial surfaces and retrocorneal plaques, while these boundaries were unclear in fungal keratitis [10]. In our current study, ASOCT findings in both of the cases with retrocorneal plaques (Cases #2 and #3) showed clear boundaries, which demonstrated that these were not similar to fungal keratitis.

Topical corticosteroids were effective for the treatment of this disease, as the stromal feathery infiltration and inflammation of the endothelium and anterior chamber were immediately suppressed after the addition of topical steroids in all cases. We additionally used oral valacyclovir in all cases and topical acyclovir in Cases #2 and #3. It was not clear, however, which of these, the oral antivirals, the topical antivirals, or both, would be the most appropriate for treating HSV diffuse endotheliitis. In future, it is expected that there will be a further accumulation of PCR-confirmed HSV diffuse endotheliitis cases.

This report has a limitation that the possibilities of fungal co-infection were not completely excluded, because these cases had been empirically treated by antifungals for a couple of weeks. However, multiplex PCR did not detect any of fungal DNA including fungal 18S rRNA. Second, fungal keratitis especially by filamentous fungi is refractory and usually difficult to cure by antifungal medication for a short period of time. Thus, we believe that these cases were not fungal co-infection. If confocal microscopy was available in our department, it might have been useful to exclude fungal infection.

In conclusion, this report described 3 cases of HSV diffuse endotheliitis, which is a rare form of HSK. All 3 cases were successfully diagnosed through the use of real-time PCR in combination with direct microscopy. Topical corticosteroids in conjunction with oral and topical antivirals were also shown to be effective in the treatment of HSV diffuse endotheliitis.

## Data Availability

The datasets used and/or analysed during the current study are available with the corresponding author on reasonable request.

## Abbreviations

HSK: Herpes simplex keratitis
PCR: Polymerase chain reaction
BCVA: Best corrected visual acuity
IOP: Intraocular pressure
KP: Keratic precipitate
GAPDH: Glyceraldehyde 3-phosphate dehydrogenase
HSV-1: Herpes simplex virus type 1
ASOCT: Anterior segment optical coherence tomography
